# A Real-Life Study in Sequential Therapy for Severe Menopausal Osteoporosis

**DOI:** 10.3390/jcm14020627

**Published:** 2025-01-19

**Authors:** Oana-Claudia Sima, Mihai Costachescu, Mihaela Stanciu, Claudiu Nistor, Mara Carsote, Denisa Tanasescu, Florina Ligia Popa, Ana Valea

**Affiliations:** 1 PhD Doctoral School of “Carol Davila”, University of Medicine and Pharmacy, 010825 Bucharest, Romania; oana-claudia.sima@drd.umfcd.ro; 2 Department of Radiology and Medical Imaging, Fundeni Clinical Institute, 022328 Bucharest, Romania; mihai.costachescu@drd.umfcd.ro; 3 Thoracic Surgery Department, “Dr. Carol Davila” Central Emergency University Military Hospital, 010825 Bucharest, Romania; 4 Department of Endocrinology, “Lucian Blaga” University of Sibiu, Victoriei Blvd., 550024 Sibiu, Romania; 5 Department of Endocrinology, Clinical County Emergency Hospital, 550245 Sibiu, Romania; 6 Department 4—Cardio-Thoracic Pathology, Thoracic Surgery II Discipline, “Carol Davila” University of Medicine and Pharmacy, 050474 Bucharest, Romania; 7 Department of Endocrinology, “Carol Davila” University of Medicine and Pharmacy, 020021 Bucharest, Romania; carsote_m@hotmail.com; 8 Medical Clinical Department, Faculty of Medicine, “Lucian Blaga” University of Sibiu, 550169 Sibiu, Romania; denisa.tanasescu@ulbsibiu.ro; 9 Department of Physical Medicine and Rehabilitation, Faculty of Medicine, “Lucian Blaga” University of Sibiu, 550024 Sibiu, Romania; florina-ligia.popa@ulbsibiu.ro; 10 Department of Endocrinology, “Iuliu Hatieganu” University of Medicine and Pharmacy, 400012 Cluj-Napoca, Romania; ana.valea@umfcluj.ro; 11 Department of Endocrinology, County Emergency Clinical Hospital, 400347 Cluj-Napoca, Romania

**Keywords:** osteoporosis, teriparatide, parathormone, bone, fracture, DXA, bone turnover marker, calcium, osteocalcin

## Abstract

**Background:** Teriparatide (TPT) acts against severe primary (postmenopausal) osteoporosis (MOP), and it requires continuation with another anti-resorptive drug to conserve or enhance the effects on fracture risk reduction. **Objective:** To analyse the sequential pharmacotherapy in MOP who were treated upon a 24-month daily 20 µg TPT protocol (24-mo-TPT) followed by another 12 months of anti-resorptive drugs (12-mo-AR) amid real-life settings. **Hypotheses:** 1. TPT candidates had a more severe fracture risk profile versus those who did not fulfil the TPT criteria according to the national protocol of TPT initiation; 2. Patients treated with TPT improved their DXA profile after 24 mo; 3. After 1 year of therapy since the last TPT injection, the improved bone profile and fracture risk at the end of the TPT protocol were conserved; 4. The mineral metabolism assays and fracture risk status were similar at TPT initiation between those who finished the 24 mo protocol and those who prematurely stopped it. **Methods:** This was a longitudinal, retrospective, multicentre study in MOP. The entire cohort (group A) included the TPT group (B) versus the non-TPT group (non-B). Group B included subjects who finished 24-mo-TPT (group P) and early droppers (ED), and then both continued 12-mo-AR. **Results:** Group B (40.5%) from cohort A (N = 79) vs. non-B had lower T-scores, increased age and years since menopause. A similar profile of demographic features, BTM, and prevalent fractures (73%, respectively, 57%) was found in group P (72%) vs. ED (21.8%). Group P: osteocalcin was statistically significantly higher at 12 mo (+308.39%), respectively, at 24 mo (+171.65%) vs. baseline (*p* < 0.001 for each), while at 12-mo-AR became similar to baseline (*p* = 0.615). The cumulative probability of transient hypercalcemia-free follow-up of protocol had the highest value of 0.97 at 6 mo. An incidental fracture (1/32) was confirmed under 24-mo-TPT. BMD had a mean percent increase at the lumbar spine of +8.21% (*p* < 0.001), of +12.22% (*p* < 0.001), respectively, of +11.39% (*p* < 0.001). The pharmacologic sequence for 12-mo-AR included bisphosphonates (24.24% were oral BP) or denosumab (13%). BTM showed a suppression at 12-mo-AR (*p* < 0.05), while all BMD/T-scores were stationary. No incidental fracture was registered during 12-mo-AR. **Conclusions:** All research hypotheses were confirmed. This study in high-risk MOP highlighted an effective sequential pharmacotherapy in reducing the fracture risk as pinpointed by BMD/T-score measurements and analysing the incidental fractures profile.

## 1. Introduction

Teriparatide (TPT), a bone anabolic agent, acts against osteoporosis, particularly primary and glucocorticoid-induced types [1,2,3]. In daily practice, TPT candidates display a high risk of fragility fractures, and the choice of TPT is due to an efficient risk reduction with regard to vertebral and non-vertebral osteoporotic fractures [4,5,6]. TPT, as well as abaloparatide, are parathormone (PTH)-derivate molecules, and together with romosozumab (an anti-sclerostin agent), represent the bone-forming agents amid the modern medical era in the field of osteoporosis [7,8,9]. TPT may be prescribed only once across life span according to an 18-month or 24-month protocol (subcutaneously, 20 µg per day), depending on the country protocol [10,11,12].

TPT candidates require a meticulous evaluation before drug exposure since a complex panel of co-morbidities needs to be ruled out before starting the medication, such as primary hyperparathyroidism or bone metabolic diseases [13,14,15]. Moreover, if one patient stops the drug even for a short period of time, TPT cannot be resumed; hence, the patient’s education and adherence to recommendations are essential for the overall success of the therapy [16,17,18]. Moreover, following the TPT protocol, another anti-osteoporotic drug, such as bisphosphonates or denosumab, is mandatory to immediately continue the TPT sequence in order to conserve and even enhance the benefits of prior TPT exposure [19,20,21]. The long-standing use of specific pharmacologic therapy in osteoporosis requires a complex approach that raises the concern of adverse effects while stopping one drug, a rebound phenomenon might be found after some drugs. Sequential rather than combined treatments represent the choice nowadays in most patients with osteoporosis, particularly in cases with high-fracture risk (as, for instance, calculated by FRAX) or severe (complicated) osteoporosis (in terms of prevalent osteoporotic fractures). Long-term follow-up according to a guideline-based and tailored intervention is mandatory [19,20,21].

Our objective was to analyse the sequential pharmacotherapy in menopausal patients with severe osteoporosis who were treated upon a 24-month daily TPT protocol followed by another 12 months of anti-resorptive therapies amid real-life settings.

The working hypotheses include:

1. TPT candidates had a more severe fracture risk profile versus those who were referred at the same hospitals for osteoporosis management and did not fulfil the TPT criteria according to the national protocol of TPT initiation.
2. Patients treated with TPT improved their DXA profile after finishing 24 months of exposure.
3. After another year of therapy since the last TPT injection was administered (sequence in anti-osteoporosis approach) while the patients were under anti-resorptive drugs, the improved bone profile and fracture risk at the end of the TPT protocol were conserved.
4. The mineral metabolism assays and fracture risk status were similar at TPT initiation between those who actually finished the 24-month protocol and those who prematurely stopped it.

## 2. Material and Methods

### 2.1. Study Design

This was a real-life study of longitudinal, retrospective, multicentre type, from January 2019 until January 2023. This was a sub-analysis of the PRECES study, a single-country initiative in the field of endocrinology and connected domains (“Parameters of Romanian patients with Endocrine Conditions with or without Endocrine Surgery: real-world-evidence and retrospective study”).

### 2.2. Studied Population and Protocol

**Figure 1:**
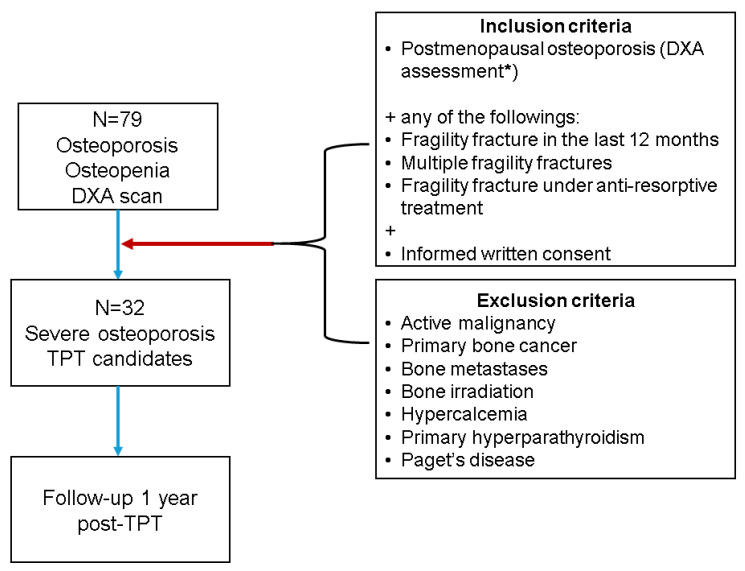
Initial study protocol with inclusion and exclusion criteria for starting teriparatide against severe osteoporosis in menopausal females (Abbreviations: N = number of patients; TPT = teriparatide) [22,23,24]; * means lowest T-score at central DXA of at least −2.5 SD at any of the central sites: lumbar spine, femoral neck or total hip.

Menopausal patients with osteoporosis and osteopenia were included, as confirmed by a central DXA (Dual-Energy X-Ray Absorptiometry) assessment (GE Lunar Prodigy device). Among this group, TPT sub-group was defined as TPT candidates according to the national protocol of TPT-free reimbursement for severe osteoporosis [22,23,24] (Figure 1).

The patients were hospitalised in each centre (university hospitals) in order to initiate TPT therapy for 2 years, and an annual evaluation was mandatory during these 24 months and in cases that experienced side effects that were considered to be related to the TPT protocol. While the decision was based on the national TPT criteria [22,23,24], each patient was followed by the current physician and the final DXA analysis was re-checked by an external radiologist (dr. M.Cos.). The entire cohort (N = 79), also named group “A”, included the TPT group (named group “B”, N = 32 patients) and group non-B (N = 47 individuals that were patients with osteoporosis or osteopenia who did not meet the criteria of TPT initiation, but they were referred to the same hospitals for fracture risk intervention).

**Figure 2:**
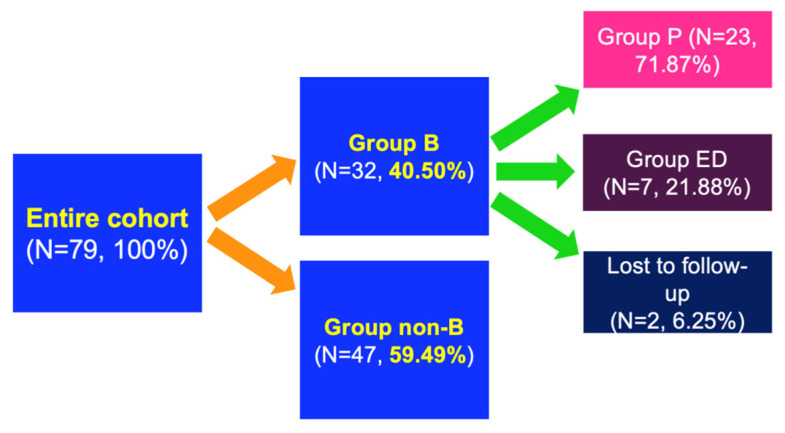
Studied subgroups from the entire cohort (group B = patients who were treated with TPT according to the 24-month national protocol; group non-B = patients who did not meet the criteria of severe osteoporosis in order to become TPT candidates; group P = patients treated with TPT for 24 months according to the standard protocol; group ED = patients who started TPT protocol, but finished it before the 24 months due to side effects); (Abbreviations: N = number of patients; P = protocol; ED = early droppers).

Further on, the analysis was focused on the sequential therapy for group B meaning group P (patients who finished the 24-month TPT protocol, N = 23 persons), and group ED (early droppers, N = 7 patients who prematurely stopped TPT), as well as another two patients who were lost to follow-up. Both groups (P and ED) continued medication against osteoporosis for another 12 months, and the results were analysed according to this post-TPT sequence, too [25]. The decision to choose the drug after TPT was stopped was individual (based on the current physician’ judgment according to the standard national and international guidelines for osteoporosis, independently of the reimbursement protocol for each anti-osteoporotic drug) (Figure 2).

The collected parameters included demographic data such as age, years since menopause, body mass index (BMI), co-morbidities, history of osteoporosis and prior medication, central DXA assessment in terms of bone mineral density (BMD), T-score, and Z-score for lumbar spine, femoral neck, and total hip, mineral metabolism assays (total and ionic serum calcium, serum phosphorus, and 24 h urinary calcium), bone hormones (PTH and 25-hydroxyvitamin D), as well as blood turnover markers (of formation: osteocalcin, P1NP, alkaline phosphatase, and of resorption: CrossLaps). Each patient underwent a screening profile X-ray of the thoracic–lumbar spine at baseline and after each year of therapy. These captures were re-assessed amid a second (independent) radiological analysis for this study (dr. M.Cos.). Prior fractures (before current admission) were registered in the patients’ medical records as osteoporotic (fragility fractures). The screening X-ray was performed annually under TPT protocol and one year after finishing it.

### 2.3. Statistical Analysis

Data analysis was performed using Excel 16.90.2 (Microsoft, Redmond, WA, USA) and SPSS 29.0.2.0 (SPSS, Inc., Chicago, IL, USA). The normality of continuous variables was assessed with the Kolmogorov–Smirnov test. Central tendencies were reported as mean ± standard deviation (SD) for normally distributed data and as quartiles (Q1, median/Q2, and Q3, respectively, IQR or interquartile interval) for non-normally distributed data. Associations between categorical variables were evaluated using the chi-squared test or Fisher’s exact test when appropriate. For comparison of continuous variables, Student’s *t*-test was used, and if assumptions of normality were significantly violated, the Mann–Whitney U test was applied alternatively. Cumulative probability curves were generated using the Kaplan–Meier method. Statistical significance was defined as a *p*-value < 0.05.

### 2.4. Ethical Aspects

Each subject signed an informed consent during hospitalisation in each university centre/hospital according to the local protocol. The retrospective data analysis was approved by the local Ethical Committees (number; number 665 from 31 January 2024; number 124 from 25 June 2024; number 6284 from 8 February 2024; number 2058 from 30 January 2024).

## 3. Results

### 3.1. Analysis at TPT Initiation: Entire Cohort (Containing Group B Versus Group Non-B)

A total of 79 postmenopausal women (designated as “entire cohort” or group “A”) with osteoporosis were analysed. A total of 40.51% of the patients from this initial cohort were offered TPT (group B), according to the national protocol [22,23,24]. Age was statistically significantly higher in group B (of 66.50 ± 9.05 years) compared to non-B (of 62.23 ± 7.82 years, *p* = 0.028), as well as time since menopause: 21.16 ± 10.23 years versus 14.77 ± 9.73 years (*p* = 0.006) (Table 1 and Table A1).

Lumbar BMD and T-score were statistically significantly lower in group B compared to non-B (*p* < 0.001), as well as found at the femoral neck (*p* < 0.001), respectively, total hip BMD (*p* < 0.001) (Table 2).

### 3.2. Patients Treated with TPT (Group B): 24-Month Protocol (Group P) Versus Early Droppers (Group ED)

A total of 71.87% of the subjects in group B finished the 24-month protocol of TPT and presented for follow-up (group P), while 21.88% were early droppers (group ED) in addition to 6.25% of the individuals from group B who were lost to follow-up. Group P had similar features with ED at baseline (Table 3).

The prior mentioned parameters of the mineral metabolism were similar between group P and ED, as well as 25-hydroxyvitamin D, parathormone (*p* = 0.970, respectively, *p* = 0.204), and bone turnover markers (Table A2). Lumbar DXA analysis revealed a statistically significant lower T-score in group P versus ED (Table 4).

86.96% of the females in group P (20/23) and 100% of the subjects in group ED (7/7) were non-responders to prior anti-resorptive (at TPT initiation), this being defined in lower BMD score at serial DXA (“DXA non-responder)” or incidental osteoporotic fracture under anti-osteoporotic medication (“fracture non-responder”) or both (“DXA + fracture non-responder”) as shown in Table 5.

In group ED, patients followed TPT between 1.00 and 13.00 with a mean of 8.71 ± 4.75, and a median of 12.00 (IQR 6.00, 12.00). The side effects are shown below. One patient was considered non-responder to TPT due to an incident fragility (vertebral) fracture after the first 12 months of TPT exposure that represented an indication of stopping the drug according to the standard TPT protocol [22,23,24] (Table 6).

### 3.3. Sequential Pharmacotherapy: First 12 Months Following the TPT Protocol (Group P Versus Group ED)

Group P showed that ionised serum calcium was statistically significantly lower one-year post-TPT compared to the value at the end of the 24-month TPT protocol (*p* = 0.002), as PTH (*p* = 0.009). Moreover, serum bone turnover markers showed a statistically significant decrease one-year post-TPT protocol versus the values at the end of the 24-month TPT protocol (*p* < 0.05 for each biomarker). In group ED, mineral metabolism assays were all similar between the moment of the last TPT injection and one year later (Table 7).

**Figure A1:**
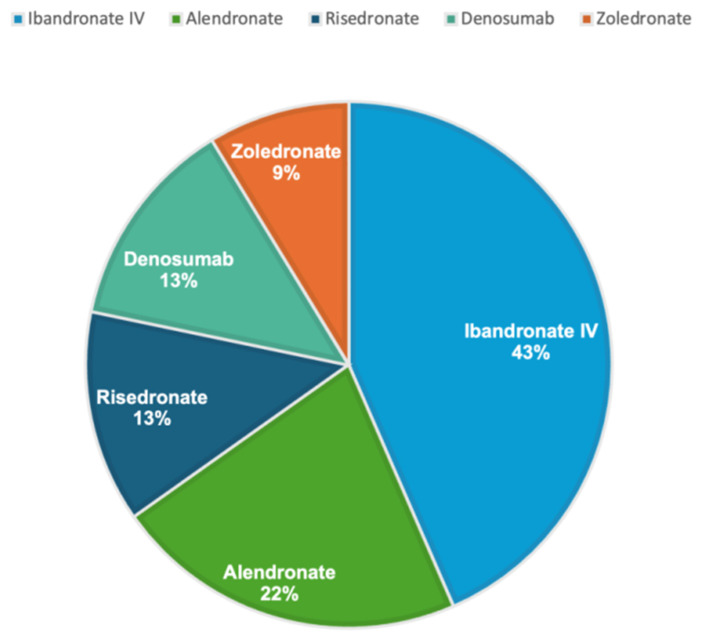
Pie chart showing the pharmacologic approaches following TPT protocol.

The values of BMD and T-score at central DXA were stationary for one year since the last TPT injection (Table A3). Post-TPT sequence included the following drugs: 43.00% of the individuals were treated with ibandronate, 22.00% with alendronate, 13.00% with risedronate, 13.00% with denosumab, and 9.00% with zoledronate (Figure A1). A total of 24.24% of the women in group P continued with oral bisphosphonates after finishing TPT and 36.36% continued with intravenous bisphosphonates. Bone formation and resorption markers showed a statistically significant suppression after one year of bisphosphonates (since TPT was stopped) for both sub-groups (oral and intravenous treatment; *p* < 0.05 for each) (Table A4). Lumbar, femoral neck, and total hip BMD and T-score were conserved after one year of oral or intravenous bisphosphonates with reference to the level at TPT stop (Table A5).

### 3.4. A Sub-Analysis Between 24 Months into TPT Protocol Versus One Year Post-TPT Protocol (Only the Patients Who Finished the Entire Protocol)

Group P had higher ionised calcium mean values at 12 months (4.31 ± 0.30 mg/dL) and at 24 months (4.28 ± 0.20 mg/dL) into TPT protocol compared to the baseline assays (4.03 ± 0.34 mg/dL; *p* = 0.017, respectively, *p* = 0.049), and increased serum phosphorus at 12 months of TPT protocol compared to the first evaluation at TPT initiation (3.56 ± 0.52 mg/dL versus 3.44 ± 0.60 mg/dL, *p* = 0.048) (Table A6).

**Figure 3:**
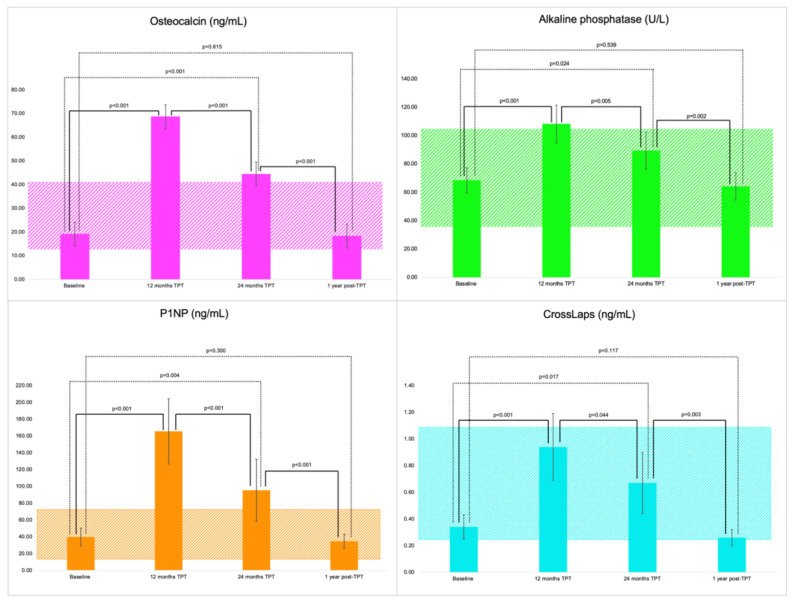
Bar charts showing the mean values for bone turnover markers in group P (N = 23) at baseline, during TPT treatment (after 12 months, respectively, after 24 months), and one-year post-TPT protocol (with 95% confidence interval).

Bone turnover markers under TPT exposure showed a typical response to this bone-forming agent in terms of osteocalcin, which was statistically significantly higher at 12 months, respectively, at 24 months versus baseline (*p* < 0.001 for each), while one year after finishing the TPT protocol osteocalcin became similar to the baseline value (*p* = 0.615). Alkaline phosphatase was higher at 12 months at 108.39 ± 26.71 IU/L and at 24 months at 89.60 ± 23.50 IU/L versus baseline assays (*p* < 0.001, respectively, *p* = 0.024), while 12 months after the last TPT injection, it decreased to 64.31 ± 21.38 IU/L, similar to the baseline level (*p* = 0.539). P1NP statistically significantly increased during the first 12 months of TPT to 151.00 ng/mL (IQR 137.00, 205.50) (*p* < 0.001), respectively, at the end of the 24-month protocol, of 75.00 ng/mL (IQR 51.08, 96.54) (*p* = 0.004) compared to the baseline value, while one-year post-TPT, the median of 27.65 ng/mL (IQR 23.96, 42.63) was similar to the baseline level (*p* = 0.300). CrossLaps at 12 months (mean of 0.95 ± 0.53 ng/mL) and at 24 months of TPT exposure (mean of 0.67 ± 0.50 ng/mL) were statistically significantly higher than baseline (*p* < 0.001, respectively, *p* = 0.017), and after one-year post-TPT, the value decreased similar to the baseline (*p* = 0.117) (Figure 3).

**Figure 4:**
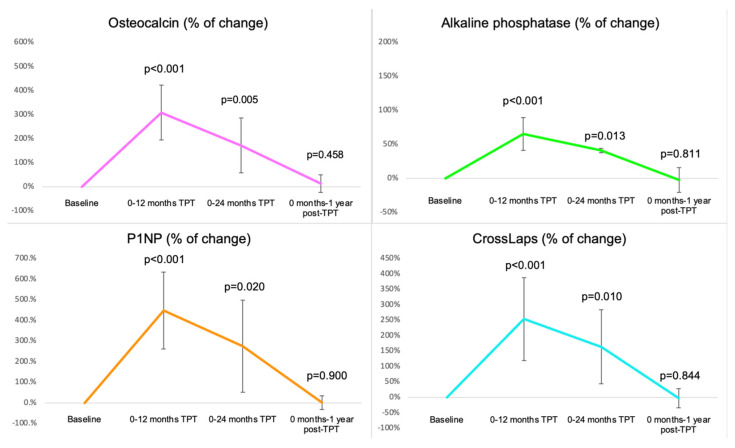
Line charts showing the mean percentage change in bone turnover markers in the group treated with TPT according to the 24-month protocol relative to the baseline values (with 95% confidence interval) (Abbreviations: TPT = teriparatide).

In terms of the percent change, osteocalcin statistically significant increased with +308.39% (*p* < 0.001), respectively, with +171.65% (*p* = 0.005); alkaline phosphatase increased with +65.48% (*p* < 0.001), respectively, +41.14% (*p* = 0.013); P1NP increased with +448.15% (*p* < 0.001), respectively, with +275.20% (*p* = 0.020); CrossLaps increased with +253.89% (*p* < 0.001), respectively, +163.90% (*p* = 0.01), at 12, respectively, 24 months into TPT protocol compared to the baseline (Figure 4).

**Figure 5:**
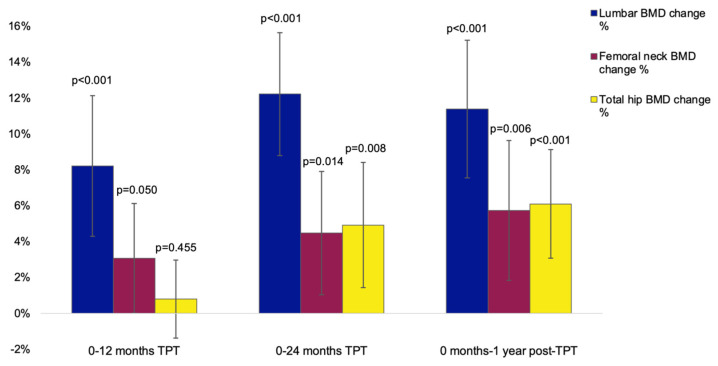
Bar charts showing the mean percentage change in DXA-BMD at central DXA sites in the TPT group (N = 23) relative to the baseline values (with 95% confidence interval).

**Figure A2:**
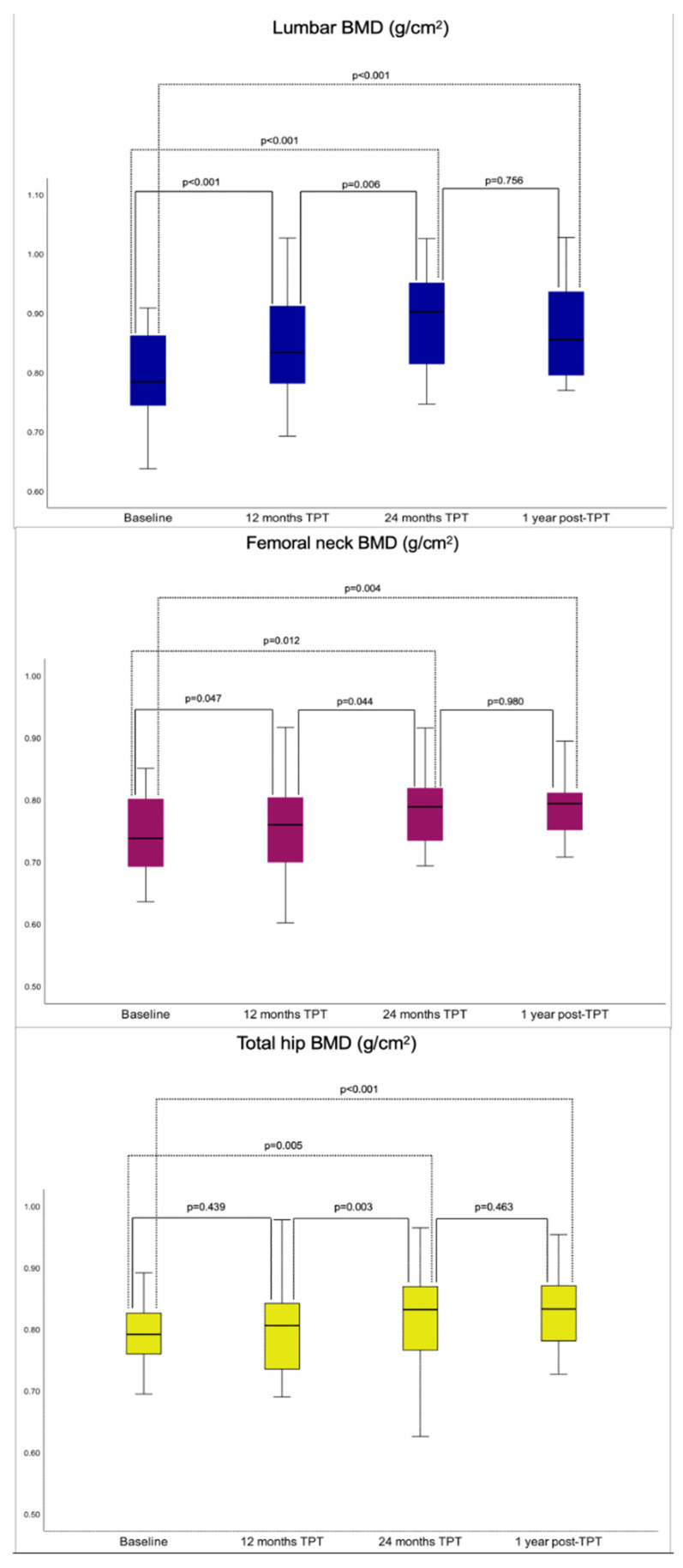
Boxplots showing the distributions of lumbar, femoral neck, and total hip DXA-BMD in the treated with TPT for 24 months: at baseline, during TPT treatment (at 12 months and 24 months), and one year after the TPT protocol (Abbreviations: BMD = bone mineral density; TPT = teriparatide, y = year).

Lumbar DXA parameters (BMD and T-scores) were statistically significantly higher after 12 months, 24 months, and 12 months following the TPT protocol versus the first evaluation (*p* < 0.001 for each) (Table A7, Figure A2). BMD showed a mean percent increase at the lumbar spine of +8.21% (*p* < 0.001), +12.22% (*p* < 0.001), respectively, +11.39% (*p* < 0.001), at the femoral neck of +3.07% (*p* = 0.050), +4.47% (*p* = 0.014), respectively, +5.74% (*p* = 0.006) from baseline versus 12, 24 months of TPT, respectively, 12 months post-TPT. No incidental fracture was registered within the first year post-TPT and amid annual evaluation at the end of 12 mo post-TPT (Figure 5).

### 3.5. A Sub-Analysis of Transitory Hypercalcemia During 24-Month TPT Protocol

**Figure 6:**
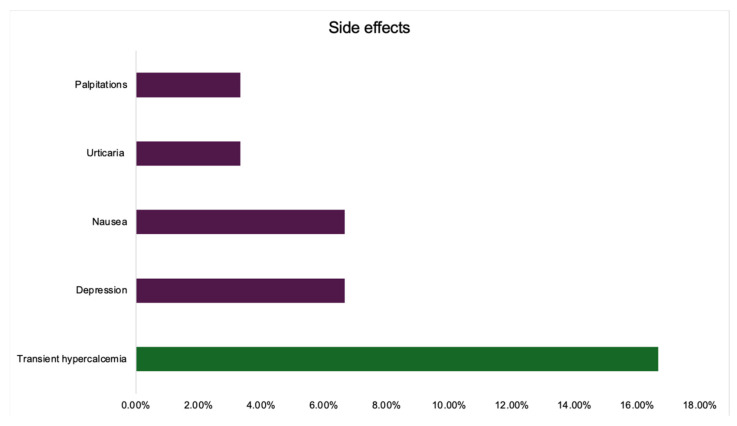
Bar chart showing the percentage of adverse reactions during TPT treatment.

**Figure A3:**
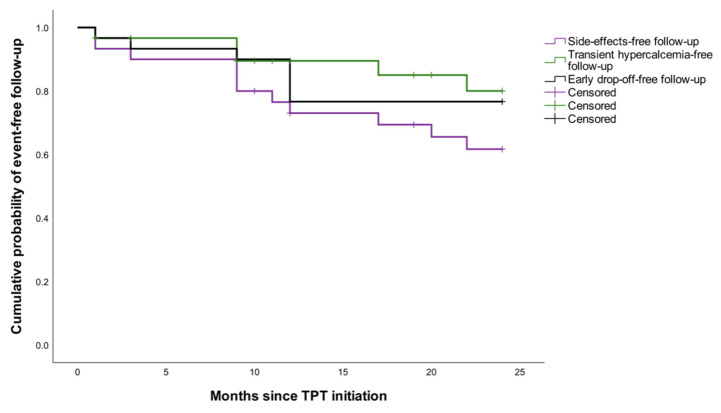
Kaplan–Meier curve showing the cumulative probabilities of side effects-, early drop-off- and hypercalcemia-free follow-up in groups P and ED (N = 30).

A total of 15.63% of the menopausal females experienced transient hypercalcemia during TPT exposure. No case was symptomatic (Figure 6 and Figure A3).

Transient hypercalcemia appeared after a mean period of 12.60 ± 8.62 months (Table 8).

Group P was re-divided into two sub-groups: 16.67% of the patients experienced transient hypercalcemia (group PCa) and 83.33% of the osteoporotic females did not present this biochemical effect throughout the 24-month TPT treatment (group non-PCa). All the mentioned variables were similar between group PCa and non-PCa at the moment when TPT was initiated (Table A8).

The cumulative probability of side effects-free follow-up of protocol and early drop-off-free follow-up of protocol decreased over time after TPT initiation showed: at 6 months, the probability was 0.90 ± 0.06, and 0.93 ± 0.05, respectively; at 12 months, the probability declined to 0.73 ± 0.08, and 0.90 ± 0.06, respectively. At 18 months, both probabilities converged, with side effects-free and early drop-off-free follow-up of protocol, each reaching 0.67 ± 0.08 and 0.77 ± 0.08. At 24 months, the cumulative probability for side effects-free follow-up of the protocol was reduced to 0.62 ± 0.09 and for early drop-off-free remained 0.77 ± 0.08. The cumulative probability of transient hypercalcemia-free follow-up of protocol had the highest value of 0.97 ± 0.03 at 6 months, declined to 0.90 ± 0.06 at 12 months and remained stationary at 18 months, followed by a decrease to 0.80 ± 0.08 at 24 months (Table A9, Figure A2).

## 4. Discussion

All the working hypotheses were confirmed. We introduced a cohort regarding a pharmacological sequence in TPT candidates: each menopausal osteoporotic female became her own control at 12 months, respectively, at 24 months into the protocol of daily, subcutaneous 20 µg of TPT, and then at 12 months under anti-resorptive medication (post-TPT). Of note, the pharmacologic intervention was retrospectively analysed (observational study) according to the prior therapy that was applied based on the national guidance and protocol of prescription, but a case-by-case strategy was decided by the subjects’ physicians regarding the drug initiation and withdrawal (if necessary), as well as the decision of starting a second anti-osteoporotic medication when TPT was no longer offered to the patient (either due to finishing the 2-year protocol or due to side effects that were considered to be related to the TPT administration, as seen in group ED).

Generally, TPT is indicated in selected cases of osteoporosis with a severe profile of high fracture risk or non-responders to prior anti-resorptive drugs or even contraindications to other agents against osteoporosis; that is why the current sample size might not be impressive amid a multi-centric collection of data [22,23,24]. Also, a meticulous selection considering various exclusion criteria was mandatory for the TPT initiation [22,23,24,25,26,27,28], and these restricted the number of subjects in daily practice (Figure 1). Yet, TPT candidates (group B) represented 40.51% of the entire cohort of post-menopause women who were referred to as inpatients in order to decide the pharmacologic intervention of fracture risk reduction. Group A included adults who required osteoporosis management at a hospital since their primary care physicians decided not to follow them as outpatients due to a complex panel of co-morbidities or a history of complicated osteoporosis.

Real-world management of high-risk osteoporotic patients showed that currently, approximately 20% of them receive non-bisphosphonates drugs upon a risk- and guideline-based strategy and TPT represents one option among these medications [29]. However, these data are highly variable depending on each country’s protocol and free reimbursement. We identified a statistically significant increased age and duration since menopause in TPT candidates versus non-TPT group (of 66.50 ± 9.05 versus 62.23 ± 7.82 years, *p* = 0.028, respectively, 21.16 ± 10.23 versus 14.77 ± 9.73 years; *p* = 0.006), while BMI and the panel of cardio-metabolic co-morbidities (affecting up to half of the subjects) was similar between these groups. Of note, the co-presence of metabolic ailments does not restrict TPT administration [22,23,24,30,31,32,33]. Moreover, group B presented statistically significant higher 25-hydroxyvitamin D levels than the non-TPT group at drug initiation, and this is explained by the fact that, according to the protocol and guideline recommendations, the subjects who are offered TPT need to have an efficient correction of vitamin D deficiency before and during protocol [22,23,24,25] and this explains the steady values across the entire study. Moreover, the bone turnover markers profile was suppressed in the TPT group versus the non-TPT group at baseline; this is due to prior anti-resorptive exposure in group B. As expected for the patients with severe osteoporosis, BMD and T-score at all central DXA sites were statistically significantly lower in TPT candidates than found in group non-B.

A total of 72% of the subjects successfully finished the 24-month protocol (group P). One explanation for the 6.25% of the females who were lost to follow-up might be related to the potential adherence issues amid the COVID-19 pandemic [34]. Of note, the profile of demographic features, co-morbidities, mineral metabolism assays, and bone turnover markers was similar at TPT initiation between group P and ED (the median period until the early TPT withdrawal was 12 months); thus, we might conclude that they are not relevant to pinpoint the early droppers from the start. Notably, the rate of prevalent fractures was similarly high (between 73% and 57%), while only the lumbar T-score was statistically significantly lower in group P versus ED: −3.33 ± 0.63 versus −2.58 ± 0.83 SD (*p* = 0.043), and the rate of pre-TPT exposure to bisphosphonates was of 86.93%, respectively, 100%. Additionally, transient hypercalcemia was experienced by 15.63% of the females during the TPT protocol (without causing the drug withdrawal in any case) after a mean period of 12.60 ± 8.62 months. While this particular pharmacodynamics of the serum calcium has been reported under TPT in certain patients, the pathogenic mechanisms of this effect are less understood so far, neither the constellation of potential contributors, as we identified a similar mineral, demographic, and DXA profile in these subjects versus those who did not experience it [34,35,36,37,38,39,40].

As a potential bias, the calcium assays were performed yearly according to the protocol or personalised (if the current physician or primary health care physician decided additional blood assays). Thus, asymptomatic hypercalcemia might not have been registered for each case. The cumulative probability of transient hypercalcemia-free follow-up of protocol had the highest value of 0.97 ± 0.03 at 6 months. Overall, the cumulative probability of side effects-free follow-up of protocol and early drop-off-free follow-up of protocol decreased over time: (at 6 months) 0.90 ± 0.06 and 0.93 ± 0.05, respectively; (at 12 months) 0.73 ± 0.08 and 0.90 ± 0.06; and (at 18 months) of 0.67 ± 0.08 and 0.77 ± 0.08. Mineral metabolism testing during and early after the TPT protocol showed that ionised serum calcium was statistically significantly lower one-year post-TPT compared to the value at the end of the 24-month TPT protocol (*p* = 0.002), as found for serum PTH (*p* = 0.009), with similar values for total serum calcium, phosphorus, and 25-hydroxyvitamin D.

The profile of the bone turnover markers under TPT exposure was suggestive of the expected anabolic window under a bone-forming agent [41,42], as follows: osteocalcin was statistically significantly higher at 12 months (+308.39%), respectively, at 24 months (+171.65%) versus baseline (*p* < 0.001 for each), while one year after finishing the TPT protocol osteocalcin became similar to the baseline value; (total) alkaline phosphatase increased at 12 months (+65.48%) and at 24 months (+41.14%) versus initial (*p* < 0.001, respectively, *p* = 0.024), while 12 months after last TPT injection, the marker decreased to the initial level (*p* = 0.539). P1NP had the highest percent change and statistically significantly increased during the first 12 months of TPT to (+448.15%), respectively, at the end of the 24-month TPT protocol (+275.20%) compared to the baseline value, while one-year post-TPT, P1NP became similar to the baseline level. The single blood resorption marker we investigated, namely, CrossLaps, increased at 12 months (+253.89%) and at 24 months of TPT exposure (+163.90%), values that were statistically significantly higher than baseline, and after one year into the pharmacologic sequence, the value decreased to the baseline levels.

Remarkably for a high-risk cohort, only one patient (1/32) suffered a fragility fracture under TPT (after the first year of TPT exposure) and this, according to the national TPT protocol, required the drug withdrawal (being considered a TPT “non-responder”). Different studies showed various factors that may play a role in a poor response to the treatment (other than non-compliance to the daily self-injections) such as an overly suppressed profile of the bone turnover markers at drug initiation and/or a prolonged pre-exposure to bisphosphonates, as well as the co-presence of a higher fall risk and additional contributors to the bone loss as seen in secondary osteoporosis (that might overlap to the menopausal osteoporosis). However, not all studies agree on this specific matter, and so far, there is no algorithm to indicate the patients who are at higher risk of becoming non-responsive [43,44,45].

DXA profile showed that lumbar BMD and T-score were statistically significantly higher after 12 months, 24 months, and 12 months following the TPT protocol versus the first evaluation (*p* < 0.001 for each), while they were stationary one year since the last TPT injection, thus showing the conservation of the bone mass that has been achieved during protocol (under anti-resorptive medication). BMD showed a mean percent increase at the lumbar spine of +8.21% (*p* < 0.001), of +12.22% (*p* < 0.001), respectively, of +11.39% (*p* < 0.001); at the femoral neck of +3.07% (*p* = 0.050), of +4.47% (*p* = 0.014), respectively, of +5.74% (*p* = 0.006) from baseline versus 12, 24 months of TPT, respectively, 12 months post-TPT. Total hip BMD was similar between baseline and the first annual evaluation during TPT protocol (*p* = 0.455), while after two years of TPT therapy, respectively, one-year post-TPT statistically significant increased with +4.92% (*p* = 0.008), respectively, +6.10 (*p* < 0.001) compared to the initial value. Overall, these results confirm that TPT represents an effective drug in reducing the risk of fragility fracture as pinpointed by using the BMD/T-score measurements in this study [45,46,47].

The pharmacologic sequence following TPT administration included anti-resorptive, mainly bisphosphonates (43.00% were treated with ibandronate, 22.00% with alendronate, 13.00% with risedronate, and 9.00% with zoledronate) or denosumab (13%). Only 24.24% of the patients followed an oral medication with bisphosphonates. Bone formation and resorption markers showed a statistically significant suppression after one year of bisphosphonates (since TPT was stopped) for both sub-groups (oral and intravenous treatment; *p* < 0.05 for each), while the lumbar, femoral neck, and total hip BMD and T-score were stationary regardless the patients received oral or intravenous bisphosphonates. No incidental fracture was registered during the first year post-TPT.

Notably, a long-term administration of medication against osteoporosis is mandatory in these high-risk patients, and it represents an important aspect of the overall disease burden [48,49,50]. Currently, sequential pharmacologic management represents the recommended approach and not the combined sequence. Recent data pinpointed that starting with a bone-forming agent seems more useful for long-standing fracture reduction intervention than initiating the sequence with an anti-resorptive drug in subjects with an elevated risk of osteoporotic fractures [49,50,51,52]. Globally, primary osteoporosis remains a major burden of the healthcare systems, particularly for patients at high fracture risk, and the pharmacologic sequence depends on access and local reimbursement protocols in each country [53].

As limitations of the study, we already mentioned the small cohort, but TPT is recommended only in severe osteoporosis; it can only be prescribed upon a specific protocol, and the follow-up was more difficult amid the COVID-19 pandemic and early post-pandemic years. Also, the lack of data with respect to quantifying the daily calcium and vitamin D supplementation during and after the TPT protocol was overpassed by yearly measuring the serum calcium levels as 25-hydroxyvitamin D. No other assessments than those provided by the DXA-BMD/T-score and annual screening spine X-Ray were available for the quantification of the fracture outcome (e.g., as seen after using quantitative computed tomography [54] or trabecular bone score [55,56]), but the study design was based on real-life settings. Moreover, no pre-TPT analysis of the types of osteoporotic fractures nor the specific pharmacologic intervention was available, noting that prior exposure to the anti-osteoporotic drugs was found in 84.38% of the patients. Further expansion of the study should also include the assessment of the quality of life amid daily self-administration of the drug and the evaluation of the synchronous non-pharmaceutical intervention as generally recommended for osteoporotic patients.

Currently, real-world evidence in the field of osteoporosis research provides a vast amount of data, and there is an increasing recognition of the associated analyses that might generate accurate scientific evidence, in this instance, real-world evidence. In addition to the parameters that concern the natural history of a condition, understanding the therapy effectiveness as seen in real settings and also the panel of adverse events became a valuable tool for practitioners. Moreover, the medical data might be expanded to a health-related social and economic analysis in order to provide a more complex disease burden. Moreover, real-world evidence provides insights between different countries in terms of medical records (e.g., electronic, hospital-based, patients’ self-declared records), the limits of the health care systems, and helps to understand the studied population and provides the development of methods for further interventional trials [57].

## 5. Conclusions

In this real-life, multidisciplinary, longitudinal study of sequential pharmacotherapy for women diagnosed with severe osteoporosis, we confirmed the working hypotheses: TPT candidates had a more severe fracture risk profile than non-TPT candidates; women who were offered a 2-year protocol with daily TPT followed by 1-year oral or intravenous bisphosphonates or denosumab showed an effective drug in reducing the risk of fragility fracture as pinpointed by using the BMD/T-score measurements and analysing the incidental fractures profile. The initial assessments of those who finished the protocol versus early droppers were similar. After one year since TPT, the fracture risk, as assessed in this study, was stationary. Sequential therapy proved efficient in these high-risk patients, as evaluated starting with TPT exposure.

## Figures and Tables

**Table 1:** Demographic characteristics in the entire group, group B and group non-B (Abbreviations: N = number of patients; SD = standard deviation).

**Demographic Parameters**					
**Parameter**	**Descriptive Statistics (Units)**	**Entire Sample** **(N = 79, 100%)**	**Group B** **(N = 32, 40.51%)**	**Group Non-B** **(N = 47, 59.49%)**	***p*** **-Value**
Age	Mean ± SD (years)	63.96 ± 8.55	66.50 ± 9.05	62.23 ± 7.82	**0.028**
Years since menopause	Mean ± SD (years)	17.35 ± 10.37	21.16 ± 10.23	14.77 ± 9.73	**0.006**
Body mass index	Mean ± SD (kg/m^2^)	23.90 ± 3.22	23.45 ± 3.79	24.22 ± 2.74	0.302
**Prevalent comorbidities**					
High blood pressure	N (%)	45 (57.96)	15 (48.39)	30 (63.83)	0.177
Dyslipidaemia	N (%)	40 (50.63)	13 (40.63)	27 (57.45)	0.142
Diabetes mellitus	N (%)	7 (8.86)	5 (10.64)	2 (6.25)	0.500

**Table 2:** DXA evaluation in the entire cohort, group B, and group non-B at baseline evaluation (Abbreviations: BMD = bone mineral density; DXA = Dual-Energy X-Ray Absorptiometry; N = number of patients; SD = standard deviation).

**Parameter**	**Descriptive Statistics (Units)**	**Normal Range**	**Entire Sample (N = 79, 100%)**	**Group B** **(N = 32, 40.51%)**	**Group Non-B (N = 47, 59.49%)**	***p*** **-Value**
Lumbar BMD	Mean ± SD (g/cm^2^)		0.871 ± 0.158	0.794 ± 0.081	0.919 ± 0.175	**<0.001**
Lumbar T-score	Mean ± SD (SD)	>−1	−2.42 ± 1.07	−3.21 ± 0.68	−1.93 ± 0.97	**<0.001**
Lumbar Z-score	Mean ± SD (SD)		−1.14 ± 0.99	−1.69 ± 0.82	−0.80 ± 0.93	**<0.001**
Femoral neck BMD	Mean ± SD (g/cm^2^)		0.761 ± 0.102	0.710 ± 0.081	0.796 ± 0.10	**<0.001**
Femoral neck T-score	Mean ± SD (SD)	>−1	−1.93 ± 0.77	−2.33 ± 0.65	−1.66 ± 0.74	**<0.001**
Femoral neck Z-score	Mean ± SD (SD)		−0.50 ± 0.64	−0.79 ± 0.45	−0.30 ± 0.65	**0.002**
Total hip BMD	Mean ± SD (g/cm^2^)		0.814 ± 0.12	0.742 ± 0.100	0.863 ± 0.107	**<0.001**
Total hip T-score	Mean ± SD (SD)	>−1	−1.53 ± 0.97	−2.13 ± 0.80	−1.13 ± 0.88	**<0.001**
Total hip Z-score	Mean ± SD (SD)		−0.42 ± 0.70	−0.77 ± 0.67	−0.19 ± 0.63	**<0.001**

**Table 3:** Demographic characteristics in group P (subjects who finished the 24-month TPT protocol) and group ED (early droppers from the 24-month TPT protocol); (Abbreviations: N = number of patients; SD = standard deviation).

**Parameter**	**Descriptive Statistics (Units)**	**Group P** **(N = 23, 71.87%)**	**Group ED** **(N = 7, 21.87%)**	***p*** **-Value**
Age	Mean ± SD (years)	66.13 ± 8.68	64.71 ± 10.28	0.720
Years since menopause	Mean ± SD (years)	21.35 ± 10.31	17.00 ± 8.96	0.324
Body mass index	Mean ± SD (kg/m^2^)	24.24 ± 3.85	21.14 ± 3.53	0.068
High blood pressure	N (%)	12 (54.55)	2 (28.57)	0.231
Dyslipidaemia	N (%)	10 (43.48)	2 (28.57)	0.481
Prevalent fragility fracture	N (%)	17 (73.91)	4 (57.14)	0.397

**Table 4:** DXA evaluation in group P and ED at TPT initiation (Abbreviations: BMD = bone mineral density; DXA = Dual-Energy X-Ray Absorptiometry; N = number of patients; SD = standard deviation).

**Parameter**	**Descriptive Statistics (Units)**	**Normal Range**	**Group P** **(N = 23, 71.87%)**	**Group ED** **(N = 7, 21.87%)**	***p*** **-Value**
Lumbar BMD	Mean ± SD (g/cm^2^)		0.784 ± 0.078	0.845 ± 0.112	0.186
Lumbar T-score	Mean ± SD (SD)	>−1	−3.33 ± 0.63	−2.58 ± 0.83	**0.043**
Lumbar Z-score	Mean ± SD (SD)		−1.85 ± 0.76	−1.10 ± 1.04	0.094
Femoral neck BMD	Mean ± SD (g/cm^2^)		0.726 ± 0.083	0.680 ± 0.070	0.204
Femoral neck T-score	Mean ± SD (SD)	>−1	−2.20 ± 0.66	−2.59 ± 0.53	0.171
Femoral neck Z-score	Mean ± SD (SD)		−0.81 ± 0.47	−0.79 ± 0.65	0.933
Total hip BMD	Mean ± SD (g/cm^2^)		0.747 ± 0.107	0.733 ± 0.096	0.756
Total hip T-score	Mean ± SD (SD)	>−1	−2.08 ± 0.84	−2.18 ± 0.80	0.792
Total hip Z-score	Mean ± SD (SD)		−0.82 ± 0.65	−0.72 ± 0.86	0.743

**Table 5:** Non-responders to anti-osteoporotic treatment prior to TPT exposure (N = 27/32, 84.38%); (Abbreviations: DXA = Dual-Energy X-Ray Absorptiometry; N = number of patients).

**Variable**	**Group P with Prior Therapy** **(N = 20, 86.96%)**	**Group ED with Prior Therapy (N = 7, 100%)**	***p*** **-Value**
DXA non-responder to prior treatment, N (%)	14 (70.00)	4 (57.14)	0.653
Fracture non-responder to prior treatment, N (%)	15 (75.00)	7 (100)	0.283
DXA + fracture non-responder to prior treatment, N (%)	9 (45.00)	4 (57.14)	0.678

**Table 6:** The analysis of ED group (N = 7); (Abbreviations: N = number of patients; Q = quartile; SD = standard deviation; TPT = teriparatide).

**Parameter**	**Value**
**Months of TPT exposure according to TPT protocol**	
Minimum, maximum	1.00, 13.00
Mean ± SD	8.71 ± 4.75
Median (Q1, Q3)	12.00 (6.00, 12.00)
**Side effects for early drop-off**	
Nausea, N (%)	2 (28.57)
Palpitations, N (%)	1 (14.29)
Depression, N (%)	2 (28.57)
Urticaria, N (%)	1 (14.29)
**Non-responder to TPT protocol that indicated TPT stop**	
Non-responder, N (%)	1 (14.29)

**Table 7:** Mineral metabolism assays and bone turnover markers after the last TPT injection compared to 1-year post-TPT protocol in group P and group ED (Abbreviations: BMD = bone mineral density; M = median; N = number of patients; Q = quartile; SD = standard deviation; TPT = teriparatide; y = year).

		**Group P (N = 23)**			**Group ED (N = 7)**			***p*** **-Value Between Group P and Group ED:** **1 y Post-TPT**
**Parameter**	**Descriptive Statistics (Units)**	**At TPT Stop**	**1 y Post-TPT**	***p*** **-Value**	**At TPT Stop**	**1 y Post-TPT**	***p*** **-Value**	
Total serum calcium	Mean ± SD (mg/dL)	9.69 ± 0.50	9.52 ± 0.32	0.207	9.64 ± 0.69	9.38 ± 0.50	0.417	0.666
Ionised serum calcium	Mean ± SD (mg/dL)	4.28 ± 0.20	4.11 ± 0.17	**0.002**	4.04 ± 0.24	3.92 ± 0.29	0.342	0.126
Serum phosphorus	Mean ± SD (mg/dL)	3.40 ± 0.48	3.58 ± 0.48	0.253	3.73 ± 0.44	3.62 ± 0.52	0.488	0.866
25-hydroxyvitamin D	Mean ± SD (ng/mL)	29.04 ± 9.21	35.32 ± 12.20	0.067	26.19 ± 4.97	35.45 ± 18.62	0.281	0.985
Parathormone	Mean ± SD (pg/mL)	39.72 ± 13.85	49.84 ± 16.60	**0.009**	48.31 ± 21.68	50.20 ± 19.17	0.880	0.481
Osteocalcin	M (Q1, Q3) (ng/mL)	36.00 (28.49, 46.50)	16.14 (13.28, 20.85)	**<0.001**	25.35 (20.20, 40.22)	17.65 (16.75, 21.77)	0.128	0.541
Alkaline phosphatase	Mean ± SD (U/L)	89.60 ± 23.50	64.31 ± 2 1.38	**0.002**	67.17 ± 13.48	53.18 ± 10.68	**0.032**	0.232
P1NP	M (Q1, Q3) (ng/mL)	75.00 (51.08, 96.54)	27.65 (23.96, 42.63)	**0.001**	47.11 (31.89, 76.00)	31.93 (22.81, 44.94)	0.116	0.849
CrossLaps	Mean ± SD (ng/mL)	0.67 ± 0.50	0.26 ± 0.12	**0.003**	0.40 ± 0.28	0.30 ± 0.16	0.492	0.480

**Table 8:** Timing of the transient hypercalcemia into TPT protocol.

**Months of Teriparatide Protocol**	**Value**
Minimum, maximum	1.00, 24.00
Mean ± SD	12.60 ± 8.62
M (Q1, Q3)	12.00 (9.00, 17.00)

## Data Availability

All the data are presented in this work.

## Appendix A

**Table A1:** Mineral metabolism assays and bone turnover markers in the entire initial cohort, group B, and group non-B at baseline evaluation (Abbreviations: M = median; N = number of patients; Q = quartile; SD = standard deviation).

**Parameter**	**Descriptive Statistics (Units)**	**Normal Range**	**Entire Cohort** **(N = 79, 100%)**	**Group B** **(N = 32, 40.51%)**	**Group Non-B (N = 47, 59.49%)**	***p*** **-Value**
Total serum calcium	Mean ± SD (mg/dL)	8.5–10.2	9.46 ± 0.54	9.49 ± 0.35	9.44 ± 0.64	0.701
Ionised serum calcium	Mean ± SD (mg/dL)	3.9–4.9	4.04 ± 0.35	4.05 ± 0.33	4.02 ± 0.37	0.766
24 h urinary calcium	Mean ± SD (g/24 h)	0.07–0.3	0.20 ± 0.11	0.21 ± 0.11	0.16 ± 0.13	0.519
Serum phosphorus	Mean ± SD (mg/dL)	2.5–4.5	3.60 ± 0.58	3.46 ± 0.54	3.70 ± 0.60	0.106
25-hydroyvitamin D	Mean ± SD (ng/mL)	20–100	25.13 ± 12.93	31.87 ± 12.97	20.39 ± 10.71	**<0.001**
Parathormone	Mean ± SD (pg/mL)	15–65	46.62 ± 20.04	43.11 ± 11.13	49.64 ± 25.12	0.165
Osteocalcin	M (Q1, Q3) (ng/mL)	15–46	18.58 (15.02, 27.49)	17.08 (14.45, 24.09)	23.66 (16.02, 31.77)	**0.045**
Alkaline phosphatase	Mean ± SD (U/L)	38–105	81.06 ± 35.42	67.59 ± 17.24	90.39 ± 4 1.54	**0.003**
P1NP	M (Q1, Q3) (ng/mL)	15–74	48.32 (29.59, 66.61)	32.00 (26.00, 61.00)	52.29 (44.46, 81.22)	**0.027**
CrossLaps	Mean ± SD (ng/mL)	0.226–1.008	0.41 ± 0.20	0.33 ± 0.19	0.48 ± 0.18	**0.002**

**Table A2:** Mineral metabolism assays and bone turnover markers in group P (subjects who finished the 24-month TPT protocol) and group ED (early droppers from the 24-month TPT protocol) at TPT initiation (Abbreviations: M = median; N = number of patients; Q = quartile; SD = standard deviation).

**Parameter**	**Descriptive Statistics (Units)**	**Normal Range**	**Group P** **(N = 23, 71.87%)**	**Group ED** **(N = 7, 21.87%)**	***p*** **-Value**
Total serum calcium	Mean ± SD (mg/dL)	8.5–10.2	9.51 ± 0.30	9.38 ± 0.50	0.446
Ionised serum calcium	Mean ± SD (mg/dL)	3.9–4.9	4.03 ± 0.34	4.03 ± 0.41	0.995
24 h urinary calcium	Mean ± SD (g/24 h)	0.07–0.3	0.18 ± 0.11	0.27 ± 0.10	0.218
Serum phosphorus	Mean ± SD (mg/dL)	2.5–4.5	3.44 ± 0.60	3.53 ± 0.36	0.745
25-hydroyvitamin D	Mean ± SD (ng/mL)	20–100	31.21 ± 14.00	30.98 ± 10.09	0.970
Parathormone	Mean ± SD (pg/mL)	15–65	42.11 ± 11.73	48.42 ± 9.04	0.204
Osteocalcin	M (Q1, Q3) (ng/mL)	15–46	17.16 (14.73, 21.57)	17.00 (15.49, 24.77)	0.607
Alkaline phosphatase	Mean ± SD (U/L)	38–105	68.60 ± 18.84	67.00 ± 14.02	0.861
P1NP	M (Q1, Q3) (ng/mL)	15–74	32.00 (29.11, 54.00)	44.27 (20.12, 66.61)	0.726
CrossLaps	Mean ± SD (ng/mL)	0.226–1.008	0.34 ± 0.20	0.27 ± 0.06	0.360

**Table A3:** DXA parameters after last TPT injection compared to one-year post-TPT protocol in group P and group ED (Abbreviations: BMD = bone mineral density; N = number of patients; SD = standard deviation; TPT = teriparatide; y = year; blue font = group P; green font = group ED.

		**Group P (N = 23)**			**Group ED (N = 7)**			
**Parameter**	**Descriptive Statistics (Units)**	**At TPT Stop**	**1 y Post-TPT**	***p*** **-Value**	**At TPT Stop**	**1 y Post-TPT**	***p*** **-Value**	***p*** **-Value Group P vs. ED** **1 y Post-TPT**
Lumbar BMD	Mean ± SD (g/cm^2^)	0.883 ± 0.091	0.866 ± 0.082	0.756	0.914 ± 0.114	0.915 ± 0.077	0.960	0.882
Lumbar T-score	Mean ± SD (SD)	−2.51 ± 0.78	−2.61 ± 0.70	0.916	−2.18 ± 0.90	−2.18 ± 0.62	0.999	0.989
Lumbar Z-score	Mean ± SD (SD)	−0.98 ± 0.77	−0.97 ± 0.66	0.281	−0.55 ± 0.72	−0.45 ± 0.60	0.514	0.774
Femoral neck BMD	Mean ± SD (g/cm^2^)	0.751 ± 0.095	0.757 ± 0.086	0.980	0.706 ± 0.065	0.709 ± 0.060	0.843	0.218
Femoral neck T-score	Mean ± SD (SD)	−2.04 ± 0.67	−2.04 ± 0.64	0.928	−2.40 ± 0.48	−2.33 ± 0.46	0.695	0.304
Femoral neck Z-score	Mean ± SD (SD)	−0.42 ± 0.65	−0.32 ± 0.67	0.331	−0.67 ± 0.59	−0.53 ± 0.52	0.307	0.471
Total hip BMD	Mean ± SD (g/cm^2^)	0.775 ± 0.104	0.802 ± 0.095	0.463	0.755 ± 0.089	0.737 ± 0.055	0.364	0.159
Total hip T-score	Mean ± SD (SD)	−1.85 ± 0.84	−1.63 ± 0.75	0.481	−1.98 ± 0.68	−2.14 ± 0.45	0.256	0.159
Total hip Z-score	Mean ± SD (SD)	−0.51 ± 0.75	−0.25 ± 0.70	0.169	−0.54 ± 0.84	−0.48 ± 0.55	0.749	0.499

**Table A4:** Mineral metabolism and bone turnover markers in patients who were offered oral and intravenous bisphosphonates at the end of the TPT protocol compared to one year after TPT (group P, N = 23) (Abbreviations: BP = bisphosphonates; BMD = bone mineral density; IV = intravenous; M = median; N = number of patients; SD = standard deviation; Q = quartile; brown font = oral BP; purple font = IV BP).

	**Descriptive Statistics (Units)**	**Oral BP** **(N = 8, 24.24%)**			**IV BP** **(N = 12, 36.36%)**		
**Parameter**		**At BP Initiation**	**After 1 Year of BP**	***p*** **-Value**	**At BP Initiation**	**After 1 Year of BP**	***p*** **-Value**
Total serum calcium	Mean ± SD (mg/dL)	9.52 ± 0.66	9.43 ± 0.28	0.789	9.84 ± 0.35	9.54 ± 0.38	0.051
Ionised serum calcium	Mean ± SD (mg/dL)	4.36 ± 0.28	4.08 ± 0.13	**0.046**	4.21 ± 0.14	4.11 ± 0.21	0.062
Serum phosphorus	Mean ± SD (mg/dL)	3.40 ± 0.52	3.69 ± 0.60	0.253	3.32 ± 0.57	3.47 ± 0.50	0.609
25-hydroxyvitamin D	Mean ± SD (ng/mL)	36.85 ± 8.82	38.28 ± 6.68	0.635	26.24 ± 6.73	37.65 ± 18.23	0.151
Parathormone	Mean ± SD (pg/mL)	39.93 ± 10.72	39.27 ± 11.45	0.889	35.09 ± 5.72	52.57 ± 13.38	**0.002**
Osteocalcin	M (Q1, Q3) (ng/mL)	36.00 (31.50, 47.14)	13.75 (11.68, 18.56)	**0.018**	33.90 (28.49, 44.50)	18.05 (15.25, 19.81)	**0.012**
Alkaline phosphatase	M (Q1, Q3) (ng/mL)	94.38 ± 28.56	69.73 ± 26.97	**0.014**	90.50 ± 17.14	64.00 ± 7.75	**0.040**
P1NP	M (Q1, Q3) (ng/mL)	67.50 (64.00, 120.00)	27.65 (17.17, 29.76)	**0.048**	75.00 (49.71, 103.50)	25.71 (23.21, 29.93)	**0.018**
CrossLaps	Mean ± SD (ng/mL)	0.66 ± 0.51	0.21 ± 0.04	**0.049**	0.62 ± 0.31	0.27 ± 0.14	**0.013**

**Table A5:** DXA evaluation in patients who continued for one year with oral and intravenous bisphosphonates at the end of TPT protocol (Abbreviations: BP = bisphosphonates; BMD = bone mineral density; DXA = Dual-Energy X-Ray Absorptiometry; IV = intravenous; N = number of patients; SD = standard deviation; brown font = oral BP; purple font = IV BP).

	**Descriptive Statistics (Units)**	**Oral BP** **(N = 8, 24.24%)**			**IV BP** **(N = 12, 36.36%)**		
**Parameter**		**At BP Initiation**	**After 1 Year of BP**	***p*** **-Value**	**At BP Initiation**	**After 1 Year of BP**	***p*** **-Value**
Lumbar BMD	Mean ± SD (g/cm^2^)	0.851 ± 0.098	0.840 ± 0.089	0.540	0.875 ± 0.097	0.883 ± 0.067	0.707
Lumbar T-score	Mean ± SD (SD)	−2.87 ± 0.76	−2.81 ± 0.75	0.664	−2.53 ± 0.87	−2.49 ± 0.61	0.835
Lumbar Z-score	Mean ± SD (SD)	−1.34 ± 0.81	−1.11 ± 0.72	0.103	−0.76 ± 0.72	−0.73 ± 0.53	0.830
Femoral neck BMD	Mean ± SD (g/cm^2^)	0.769 ± 0.070	0.782 ± 0.058	0.243	0.760 ± 0.113	0.762 ± 0.090	0.882
Femoral neck T-score	Mean ± SD (SD)	−1.93 ± 0.52	−1.82 ± 0.47	0.201	−2.03 ± 0.83	−2.04 ± 0.70	0.879
Femoral neck Z-score	Mean ± SD (SD)	−0.35 ± 0.48	−0.17 ± 0.50	0.048	−0.24 ± 0.76	−0.20 ± 0.56	0.718
Total hip BMD	Mean ± SD (g/cm^2^)	0.809 ± 0.107	0.825 ± 0.091	0.127	0.789 ± 0.110	0.795 ± 0.118	0.683
Total hip T-score	Mean ± SD (SD)	−1.58 ± 0.89	−1.44 ± 0.72	0.135	−1.73 ± 0.87	−1.69 ± 0.92	0.699
Total hip Z-score	Mean ± SD (SD)	−0.50 ± 0.44	−0.26 ± 0.37	0.009	−0.22 ± 0.70	−0.19 ± 0.82	0.804

**Table A6:** Mineral metabolism assays and bone turnover markers in group P (N = 23) during TPT protocol and one-year post-TPT exposure (Abbreviations: BMD = bone mineral density; M = median; N = number of patients; SD = standard deviation; Q = quartile; TPT = teriparatide; y = year).

**Parameter**	**Descriptive Statistics (Units)**	**0 Months** **TPT**	**12 Months** **TPT**	**24 Months** **TPT**	**1 y Post-TPT**	***p*** **-Value 0–12 Months**	***p*** **-Value 0–24 Months**	***p*** **-Value 12–24 Months**	***p*** **-Value 0 Months-1 y Post-TPT**	***p*** **-Value 24 Months-1 y Post-TPT**
Total serum calcium	Mean ± SD (mg/dL)	9.51 ± 0.30	9.73 ± 0.60	9.69 ± 0.50	9.52 ± 0.32	0.220	0.106	0.904	0.908	0.207
Ionised serum calcium	Mean ± SD (mg/dL)	4.03 ± 0.34	4.31 ± 0.30	4.28 ± 0.20	4.11 ± 0.17	**0.017**	**0.049**	0.911	0.537	**0.002**
Serum phosphorus	Mean ± SD (mg/dL)	3.44 ± 0.60	3.56 ± 0.52	3.40 ± 0.48	3.58 ± 0.48	**0.048**	0.781	0.354	0.373	0.253
25-hydroxyvitamin D	Mean ± SD (ng/mL)	31.21 ± 14.00	29.06 ± 11.99	29.04 ± 9.21	35.32 ± 12.20	0.325	0.220	0.646	0.327	0.067
Parathormone	Mean ± SD (pg/mL)	42.11 ± 11.73	39.14 ± 16.15	39.72 ± 13.85	49.84 ± 16.61	0.278	0.154	0.773	0.070	**0.009**
Osteocalcin	M (Q1, Q3) (ng/mL)	17.16 (14.73, 21.57)	63.00 (47.62, 97.00)	36.00 (28.49, 46.50)	16.14 (13.28, 20.85)	**<0.001**	**<0.001**	**<0.001**	0.615	**<0.001**
Alkaline phosphatase	Mean ± SD (U/L)	68.60 ± 1 8.84	108.39 ± 26.71	89.60 ± 23.50	64.31 ± 21.38	**<0.001**	**0.024**	**0.005**	0.539	**0.002**
P1NP	M (Q1, Q3) (ng/mL)	32.00 (29.11, 54.00)	151.00 (137.00, 205.50)	75.00 (51.08, 96.54)	27.65 (23.96, 42.63)	**<0.001**	**0.004**	**<0.001**	0.300	**<0.001**
CrossLaps	Mean ± SD (ng/mL)	0.34 ± 0.20	0.95 ± 0.53	0.67 ± 0.50	0.26 ± 0.12	**<0.001**	**0.017**	**0.044**	0.117	**0.003**

**Table A7:** DXA evaluation in group P during TPT protocol and 1-year post-TPT (Abbreviations: BMD = bone mineral density; M = median; N = number of patients; SD = standard deviation; Q = quartile; TPT = teriparatide; y = year).

**Parameter**	**Descriptive Statistics (Units)**	**0 Months** **TPT**	**12 Months** **TPT**	**24 Months TPT**	**1 y Post-TPT**	***p*** **-Value 0–12 Months**	***p*** **-Value 0–24 Months**	***p*** **-Value 12–24 Months**	***p*** **-Value 0 Months-1 y Post-TPT**	***p*** **-Value 24 Months-1 y Post-TPT**
Lumbar BMD	Mean ± SD (g/cm^2^)	0.784 ± 0.078	0.848 ± 0.085	0.883 ± 0.091	0.866 ± 0.082	**<0.001**	**<0.001**	**0.006**	**<0.001**	0.756
Lumbar T-score	Mean ± SD (SD)	−3.33 ± 0.63	−2.73 ± 0.76	−2.51 ± 0.78	−2.61 ± 0.70	**<0.001**	**<0.001**	**0.007**	**<0.001**	0.916
Lumbar Z-score	Mean ± SD (SD)	−1.85 ± 0.76	−1.20 ± 0.85	−0.98 ± 0.77	−0.97 ± 0.66	**<0.001**	**<0.001**	**0.014**	**<0.001**	0.281
Femoral neck BMD	Mean ± SD (g/cm^2^)	0.726 ± 0.083	0.754 ± 0.079	0.751 ± 0.095	0.757 ± 0.086	**0.047**	**0.012**	**0.044**	**0.004**	0.980
Femoral neck T-score	Mean ± SD (SD)	−2.20 ± 0.66	−2.04 ± 0.56	−2.04 ± 0.67	−2.04 ± 0.64	0.382	**0.046**	**0.002**	0.054	0.928
Femoral neck Z-score	Mean ± SD (SD)	−0.81 ± 0.47	−0.52 ± 0.52	−0.42 ± 0.65	−0.32 ± 0.67	**0.023**	**<0.001**	**0.007**	**<0.001**	0.331
Total hip BMD	Mean ± SD (g/cm^2^)	0.747 ± 0.107	0.770 ± 0.106	0.775 ± 0.104	0.802 ± 0.095	0.439	**0.005**	**0.003**	**<0.001**	0.463
Total hip T-score	Mean ± SD (SD)	−2.08 ± 0.84	−1.87 ± 0.86	−1.85 ± 0.84	−1.63 ± 0.75	0.202	**0.003**	**0.005**	**<0.001**	0.481
Total hip Z-score	Mean ± SD (SD)	−0.82 ± 0.65	−0.63 ± 0.80	−0.51 ± 0.75	−0.25 ± 0.70	0.360	**<0.001**	**0.003**	**<0.001**	0.169

**Table A8:** Patients who experienced transient hypercalcemia compared to patients without transient hypercalcemia during TPT protocol (groups P and ED, N = 30); the assessments are provided at TPT initiation (Abbreviations: BMD = bone mineral density; DXA = Dual-Energy X-Ray Absorptiometry; M = median; N = number of patients; SD = standard deviation; Q = quartile).

**Parameter**	**Descriptive Statistics (Units)**	**Group PCa** **(N = 5, 16.67%)**	**Group Non-PCa (N = 25, 83.33 %)**	***p*** **-Value**
Age	Mean ± SD (years)	61.00 ± 4.24	67.21 ± 9.06	0.200
Years since menopause	Mean ± SD (years)	16.75 ± 2.75	22.32 ± 11.09	0.338
Body mass index	Mean ± SD (kg/m^2^)	21.13 ± 3.33	24.90 ± 3.70	0.074
High blood pressure	N (%)	3.00 (60.00)	11.00 (44.00)	0.651
Dyslipidaemia	N (%)	1.00 (20.00)	11.00 (44.00)	0.622
**Mineral metabolism assays**				
Total serum calcium	Mean ± SD (mg/dL)	9.50 ± 0.08	9.51 ± 0.34	0.926
Ionised serum calcium	Mean ± SD (mg/dL)	4.07 ± 0.05	4.02 ± 0.39	0.808
Serum phosphorus	Mean ± SD (mg/dL)	3.05 ± 0.54	3.54 ± 0.59	0.149
25-hydroxyvitamin D	Mean ± SD (ng/mL)	36.42 ± 19.43	30.12 ± 13.02	0.426
Parathormone	Mean ± SD (pg/mL)	48.83 ± 5.24	40.61 ± 12.33	0.213
**Bone turnover markers**				
Osteocalcin	M (Q1, Q3) (ng/mL)	15.71 (13.25, 24.96)	17.37 (14.99, 21.57)	0.931
Alkaline phosphatase	Mean ± SD (U/L)	63.75 ± 14.98	61.81 ± 19.92	0.579
P1NP	M (Q1, Q3) (ng/mL)	31.69 (30.24, 46.50)	34.00 (26.00, 54.00)	0.989
CrossLaps	Mean ± SD (ng/mL)	0.33 ± 0.25	0.35 ± 0.20	0.902
**DXA evaluation**				
Lumbar BMD	Mean ± SD (g/cm^2^)	0.794 ± 0.102	0.782 ± 0.075	0.788
Lumbar T-score	Mean ± SD (SD)	−3.30 ± 0.76	−3.34 ± 0.62	0.918
Lumbar Z-score	Mean ± SD (SD)	−1.65 ± 1.02	−1.90 ± 0.72	0.570
Femoral neck BMD	Mean ± SD (g/cm^2^)	0.762 ± 0.081	0.718 ± 0.084	0.354
Femoral neck T-score	Mean ± SD (SD)	−2.00 ± 0.58	−2.24 ± 0.69	0.527
Femoral neck Z-score	Mean ± SD (SD)	−0.78 ± 0.17	−0.81 ± 0.52	0.892
Total hip BMD	Mean ± SD (g/cm^2^)	0.767 ± 0.077	0.743 ± 0.115	0.701
Total hip T-score	Mean ± SD (SD)	−1.93 ± 0.61	−2.12 ± 0.90	0.690
Total hip Z-score	Mean ± SD (SD)	−0.85 ± 0.19	−0.82 ± 0.72	0.931
Prevalent fractures	N (%)	4.00 (100.00)	13.00 (68.42)	0.191

**Table A9:** Cumulative probabilities of side effects-, early drop-off- and hypercalcemia-free follow-up in groups P and ED (N = 30) (Abbreviation: SE = standard error).

**Months Since TPT Initiation**	**Cumulative Probability of Side Effects-Free Follow-Up of Protocol ± SE**	**Cumulative Probability of Early Drop-Off-Free Follow-Up of Protocol ± SE**	**Cumulative Probability of Transient Hypercalcemia-Free Follow-up of Protocol ± SE**
6 months	0.90 ± 0.06	0.93 ± 0.05	0.97 ± 0.03
12 months	0.73 ± 0.08	0.90 ± 0.06	0.90 ± 0.06
18 months	0.67 ± 0.08	0.77 ± 0.08	0.90 ± 0.06
24 months	0.62 ± 0.09	0.77 ± 0.08	0.80 ± 0.08
